# The high mobility group A2 protein epigenetically silences the *Cdh1* gene during epithelial-to-mesenchymal transition

**DOI:** 10.1093/nar/gku1293

**Published:** 2014-12-09

**Authors:** E-Jean Tan, Kaoru Kahata, Oskar Idås, Sylvie Thuault, Carl-Henrik Heldin, Aristidis Moustakas

**Affiliations:** 1 Ludwig Institute for Cancer Research, Science for Life Laboratory, Uppsala University, Uppsala SE-75124, Sweden; 2 Department of Medical Biochemistry and Microbiology, Science for Life Laboratory, Uppsala University, Uppsala SE-75123, Sweden

## Abstract

The loss of the tumour suppressor E-cadherin (*Cdh1*) is a key event during tumourigenesis and epithelial–mesenchymal transition (EMT). Transforming growth factor-β (TGFβ) triggers EMT by inducing the expression of non-histone chromatin protein High Mobility Group A2 (HMGA2). We have previously shown that HMGA2, together with Smads, regulate a network of EMT-transcription factors (EMT-TFs) like Snail1, Snail2, ZEB1, ZEB2 and Twist1, most of which are well-known repressors of the *Cdh1* gene. In this study, we show that the *Cdh1* promoter is hypermethylated and epigenetically silenced in our constitutive EMT cell model, whereby HMGA2 is ectopically expressed in mammary epithelial NMuMG cells and these cells are highly motile and invasive. Furthermore, HMGA2 remodels the chromatin to favour binding of *de novo* DNA methyltransferase 3A (DNMT3A) to the *Cdh1* promoter. E-cadherin expression could be restored after treatment with the DNA de-methylating agent 5-aza-2′-deoxycytidine. Here, we describe a new epigenetic role for HMGA2, which follows the actions that HMGA2 initiates via the EMT-TFs, thus achieving sustained silencing of E-cadherin expression and promoting tumour cell invasion.

## INTRODUCTION

Epithelial-to-mesenchymal transition (EMT) is an important event which takes place during development, wound-healing and tumour progression (1). A prominent EMT feature is the downregulation of the tumour-and-invasion suppressor E-cadherin (*Cdh1*), which enables a cell to dissolve its cell–cell contacts and break away from its neighbours. Mesenchymal genes, such as N-cadherin and fibronectin, are also upregulated to facilitate invasion and migration (1). EMT is reversible and this process is termed mesenchymal–epithelial transition (MET). Tumour cells during malignancy progression re-activate various embryonic pathways including the EMT and MET programs for dissemination and metastasis, respectively (2,3).

EMT is regulated by many signalling pathways and involves a reactivation of molecular networks of transcription factors (EMT-TFs), such as members of the Snail, ZEB and Twist families that converge to regulate common sets of genes, including E-cadherin, which is transcriptionally repressed (4). The mechanisms by which EMT-TFs repress the *Cdh1* gene often involve co-repressors or epigenetic modifications on the histones or DNA (4–6). Epigenetic regulation of gene expression dynamically alters the chromatin into a closed or open conformation that is associated with repressive or active transcription, respectively. The DNA methyltransferases (DNMTs) and histone modifying enzymes are functionally linked to each other and play key roles in the remodelling of chromatin (7). DNA methylation is catalysed by DNMTs, which transfer a methyl group onto the cytosine of a CpG dinucleotide. DNMT1 is known as the maintenance DNMT that preserves the methylation pattern of genes after every cycle of DNA replication. DNMT3A and DNMT3B are *de novo* DNA methyltransferases responding to physiological signalling processes and their action mediates DNA methylation at genomic places previously lacking such modification (7). The *Cdh1* promoter is often silenced via DNA hypermethylation in breast cancers and during EMT (8–10).

Transforming growth factor (TGFβ) is a potent inducer of EMT (11). TGFβ binds its type I and II serine/threonine kinase receptors and activates the Smad2/3/4 complexes, which then accumulate in the nucleus and regulate gene transcription. TGFβ induces EMT by upregulating high mobility group A2 (HMGA2) (12). HMGA2 is a non-histone chromatin factor which contains three AT-hooks that bind to AT-rich sequences on the DNA; it modulates gene expression by remodelling of the chromatin state and influencing the binding affinities of transcription factors or other nuclear proteins for DNA (13). HMGA2 is an embryonic protein that is usually silenced in normal adult tissues. Overexpression of HMGA2 is associated with tumour growth and metastatic progression (14–16).

We have previously shown that HMGA2 interacts with Smad proteins to regulate the expression of Snail1 (here referred to as Snail) and other EMT-TFs (12,17). HMGA2 can also activate the Twist1 (here referred to as Twist) promoter and induce Twist expression (18). Stable clones of the mouse mammary epithelial NMuMG cells overexpressing HMGA2 (NM-Hmga2) mimicked a non-reversible EMT phenotype characterized by the complete loss of expression of E-cadherin at the mRNA and protein level (17,18). The depletion of Snail, or both Snail and Twist, by stable transfection of short-hairpin RNA (shRNA) in NM-Hmga2 cells, led to a reassembly of the tight junctions and into a partial MET state. However, relative silencing of these two EMT-TFs did not allow the re-expression of E-cadherin (18). We hypothesized that HMGA2, as a chromatin re-modeller, in addition to inducing key EMT-TFs like Snail and Twist, could have a role in epigenetically silencing the *Cdh1* gene during EMT. In this study, we demonstrate that aberrant HMGA2 can modulate the chromatin landscape, such that the *Cdh1* promoter becomes methylated and gains histone modifications associated with gene repression, adding another key mechanism by which a cell sheds its epithelial features and prepares for migration and invasion.

## MATERIALS AND METHODS

### Cells, transfections and reagents

Mouse mammary epithelial cells NMuMG, NMuMG overexpressing HMGA2 (NM-Hmga2) and their derivative clones expressing stably short-hairpin RNAs (shRNAs), Hmga2-sh*Snail* and Hmga2-sh*Snail*-sh*Twist*, and human embryonic kidney (HEK) 293T cells have been described previously (17,18). An empty vector-transfected clone of NMuMG (NM-mock), which has an epithelial morphology, served as control for NM-Hmga2. NMuMG and derivative stable clones were cultured in Dulbecco's modified Eagle's medium (DMEM) containing 10% foetal bovine serum and 10 μg/ml insulin, complemented with 5 μg/ml blasticidin for NM-Hmga2-sh*Snail* or NM-Hmga2–sh*Snail*-sh*Twist* clones. Lentiviral constructs expressing sh*HMGA2* (TRCN0000021966 and TRCN0000021967) and non-targeting control (shControl) were obtained from the Sigma Mission shRNA library (Sigma–Aldrich Sweden AB, Stockholm, Sweden). NM-Hmga2 cells were infected at a multiplicity of infection equal to 1 and selected with 1 μg/ml puromycin to generate extra control cells where the overexpressed HMGA2 was silenced stably with the shRNA. MCF10A derived MCF10CA1a.cl1 cells (referred to as MCF10CA1a (19)) were maintained in DMEM/F12 supplemented with 5% foetal bovine serum, 100 U/ml penicillin, and 100 μg/ml streptomycin. MDA-MB-231(-eco) cells, which express the ecotropic retrovirus receptor-internal ribosome entry site-green fluorescent protein (GFP) construct (20), were cultured in DMEM containing 10% foetal bovine serum and were used to generate stable clones with HMGA2 knocked-down. The ecotropic retrovirus-producing cells were transfected with the empty pRetroSuper vector (MDA-mock) or pRetroSuper vector coding a shRNA against human *HMGA2* (MDA-shHmga2), using the calcium phosphate precipitation method. The short-hairpin sh*HMGA2* was designed based on one of the 4 sequences included in the Dharmacon D-043585-03 *HMGA2* siRNA pool (forward 5′-AGAGGCAGACCTAGGAAAT-3′; reverse 5′-ATTTCCTAGGTCTGCCTCT-3′) (Dharmacon/Thermo Fischer Scientific Inc., Waltham, MA, USA). Cells were subsequently selected by 0.5 μg/ml puromycin.

Transient transfections of siRNAs were done using DharmaFECT1 siRNA transfection reagent (Dharmacon/Thermo Fischer Scientific Inc., Waltham, MA, USA) and siRNAs were: non-targeting siRNA control (Dharmacon ON-TARGETplus non-targeting pool D-001810-10-20), control siRNA against the luciferase reporter vector pGL2 (Dharmacon D-001100-01-20), mouse si*Dnmt1* (Dharmacon ON-TARGETplus SMART pool L-056796-01), mouse si*Dnmt3a* (Dharmacon ON-TARGETplus SMARTpool L-065433-01) and human si*HMGA2* (Dharmacon D-013495-02, D-013495-04).

The antibodies used were: mouse anti-DNMT3A, rabbit anti-Histone H3, mouse anti-Histone H3 (tri methyl K4), mouse anti-Histone H3 (acetyl K9), mouse anti-Histone H3 (tri methyl K27), rabbit anti-Histone H3 (tri methyl K9) and rabbit anti-Snail from Abcam (Cambridge, UK). Mouse anti-GAPDH was from Ambion (Life Technologies Corp., Foster City, CA, USA). Mouse and rabbit anti-CTCF were from Millipore (Merck/Millipore, Billerica, MA, USA). Mouse anti-E-cadherin and mouse anti-N-cadherin were from BD Transduction Laboratories (BD Biosciences, Stockholm, Sweden). Rabbit anti-fibronectin was from Sigma–Aldrich Sweden AB, Stockholm, Sweden. Rabbit anti-DNMT1, rabbit anti-HA, mouse and rabbit anti-HMGA2, mouse anti-Twist (Twist2C1a), control rabbit anti-IgG, control mouse anti-IgG, mouse anti-α-tubulin, rabbit anti-Pol II, secondary horse radish peroxidase-conjugated anti-mouse IgG and secondary horse radish peroxidase-conjugated anti-rabbit IgG were from Santa Cruz Inc. (Santa Cruz, CA, USA).

Recombinant mature human TGFβ1 was from BIOSOURCE Inc. (Life Technologies, Corp., Foster City, CA, USA). Small molecular weight TGFβ type I receptor kinase inhibitor SB505124 was from Sigma–Aldrich Sweden AB. The hypomethylating drug 5-aza-2'-deoxycytidine (5-aza; Sigma–Aldrich, Sweden, AB) was dissolved in 50% acetic acid and further diluted in serum-free medium before being incubated with the cells for the indicated time periods. Media with or without 5-aza were replenished daily due to the instability of 5-aza in solution. Histone deacetylase inhibitor Trichostatin A (TSA; BIOMOL International/Enzo Life Sciences, Solna, Sweden) was dissolved in dimethylsulfoxide (DMSO).

### Quantitative real-time PCR (qPCR)

Total RNA was isolated from cells using the RNeasy mini kit (QIAGEN AB, Sollentuna, Sweden) and cDNA synthesis was done using the iScript kit (Bio-Rad Laboratories AB, Solna, Sweden). Quantitative real-time polymerase chain reaction (PCR) experiments were performed with iQ SYBR Green Supermix (Bio-Rad Laboratories AB, Solna, Sweden) in triplicates, as previously described (17). Controls without reverse transcriptase (-RT) or without cDNA (water) were also included in every qPCR assay. Gene expression levels were determined by the comparative C_t_ method and using glyceraldehyde phosphate dehydrogenase (*Gapdh*) as reference. Primer sequences are listed in Supplementary Table S1.

### Transcriptomic analysis in the GOBO database

The gene expression data sets for *HMGA2* and *CDH1* in human breast cancer cells were obtained by utilizing the cell lines module of a web-based tool Gene expression-based Outcome for Breast cancer Online (GOBO) (http://co.bmc.lu.se/gobo) (21).

### GST pulldown

Purification of GST fusion proteins and GST pulldown assays were performed as described previously (17). The following hHMGA2 full-length and deletion mutants subcloned into pGEX4T1 were used: GST-hHMGA2 full-length (FL), GST-hHMGA2 N1 (aa 1–35), GST-hHMGA2 N2 (aa 1–25), GST-hHMGA2 C (aa 94–109) and GST-hHMGA2 ΔC (aa 1–83). All these constructs were described by us previously (17).

### Immunoblotting and immunoprecipitation

Total proteins extracted from cells were subjected to sodium dodecyl sulphate-polyacrylamide gel electrophoresis (SDS-PAGE) and analysed by immunoblotting as described in (12). For protein–protein interaction studies, two 15-cm dishes of HEK293T cells per condition were grown to 80% confluency and lysed in lysis buffer (25 mM 4-(2-hydroxyethyl-)-piperazine ethane sulfonic acid (HEPES) pH 7.5, 0.5% Triton X-100, 0.3 M NaCl, 1 mM MgCl_2_, 5 mM dithiothreitol, 1 mM EGTA, 20 mM β-glycerol phosphate, 1 mM Na_3_VO_4_, 10 mM NaF, 5% glycerol) containing protease inhibitor cocktail (Roche Diagnostics, Bromma, Sweden). Cell lysates were incubated with 2 μg antibody or no antibody overnight at 4°C, followed by addition of protein G sepharose beads (GE Healthcare Bio-Sciences AB, Uppsala, Sweden) and further incubation for 1 h at 4°C. The beads were washed with washing buffer (50 mM Tris–HCl pH 8, 0.1% Triton X-100, 0.5 mM MgCl_2_) and resuspended in SDS loading buffer. Bound proteins were resolved by SDS-PAGE and detected by immunoblotting. Molecular size markers are shown in kDa.

### Chromatin immunoprecipitation (ChIP)

ChIP was performed as described previously (17). Briefly, cells were plated at a density of 2 × 10^6^ (NMuMG clones) or 4 × 10^6^ (MDA-MD-231 clones) per 10-cm dish to achieve 80% confluency next day. One dish was used per ChIP reaction. Protein A or anti-mouse IgG Dynabeads (Invitrogen/Life Technologies Corp., Foster City, CA, USA) were used to couple 3 μg of antibodies overnight at 4°C. Antibody-beads were incubated with cell lysates for 6 h at 4°C, and washed with ChIP washing buffer. Samples were reverse cross-linked overnight at 65°C. DNA was purified using the QIAquick PCR purification kit (QIAGEN AB, Sollentuna, Sweden), and analysed by qPCR, and data are graphed as percentage of input DNA used for each immunoprecipitation. For re-ChIP assays, the first ChIP was performed and DNA–protein complexes were eluted by adding 10 mM dithiothreitol and incubating for 30 min at 37°C. The eluates were centrifuged and diluted with ChIP dilution buffer and prior to a second immunoprecipitation performed as described above. Each independent experiment was repeated two or three times, and the mean and SD values were calculated from triplicate samples. Primer sequences are listed in Supplementary Table S2.

### Restriction enzyme methylation assay

Genomic DNA was isolated using the AllPrep DNA/RNA Mini Kit (QIAGEN AB, Sollentuna, Sweden). Genomic DNA (250 ng) was digested with 10 units of HpaII, 20 units of MspI (both HpaII and MspI were from New England BioLabs Inc., Ipswich, MA, USA), or no enzymes in a 20-μl reaction for 6 h at 37°C. Digested DNA (12.5 ng) was used in a 20-μl PCR reaction using 0.5 μM forward and reverse primers spanning base pairs −108 to +3 of the *Cdh1* promoter (Supplementary Table S1), 0.1 mM of each dNTP, 0.05 units of Taq DNA polymerase (Sigma–Aldrich, Sweden AB) and 10× PCR buffer. PCR parameters were: 94°C for 3 min, 5 cycles of (94°C for 30 s, 50°C for 30 s, 72°C for 1 min), 5 cycles of (94°C for 30 s, 53°C for 30 s, 72°C for 1 min), 20 cycles of (94°C for 30 s, 56°C for 30 s, 72°C for 1 min) and 72°C for 5 min. PCR products were then electrophoresed on an agarose gel and visualized under a UV transilluminator and photographed with a Polaroid camera. HpaII is unable to cleave its recognition sequence CCGG when the internal cytosine is methylated and thus yields a specific PCR product if the region of interest is methylated, whereas its isoschizomer MspI cleaves regardless of the methylation status and no PCR product is obtained.

### Bisulphite sequencing

Genomic DNA was isolated and treated with sodium bisulphite using the EpiTect Bisulphite Kit (QIAGEN AB, Sollentuna, Sweden) according to the manufacturer's instructions. The bisulphite-converted DNA was amplified by PCR, using the following primers: 5′-TGGGTTAGAGTATAGTTAGGTTAGG-3′ (sense) and: 5′-AATCAAAACCCTCCACATACCTACA-3′ (antisense). The amplification product was electrophoresed on 1.5% agarose gels to confirm the correct product size of 416 base pairs. The product was then extracted from the gel using a QIAquick gel extraction kit (QIAGEN AB, Sollentuna, Sweden), and cloned into a pCR-II vector with the TA Cloning Kit Dual Promoter (Invitrogen/Life Technologies Corp., Foster City, CA, USA) according to the manufacturer's instructions. Ten colonies per cell line were picked for isolation of plasmid DNA, which was then sequenced (Eurofins, Uppsala, Sweden).

### Promoter reporter assay

The human *Cdh1* promoter construct Ecad3/luc (−1359/+125 base pairs relative to the transcriptional start site (TSS); kind gift from Eric Fearon, University of Michigan, USA) (22) was co-transfected with reporter plasmid pCMV-βGal for normalization, in NMuMG cells using Lipofectamine 2000 (Invitrogen/Life Technologies Corp., Foster City, CA, USA), and the transfected cells were stimulated with TGFβ for various time points. Alternatively, Ecad3/luc and pCMV-βGal were co-transfected with empty vector pcDNA3 or pcDNA3-HA-HMGA2 and pcDNA3-Flag-CTCF vectors in NMuMG cells using the same protocol. The pcDNA3-HA-HMGA2 and pcDNA3-Flag-CTCF vectors were previously reported by us (17,23). The enhanced luciferase assay kit from BD Pharmingen (Life Technologies Corp., Foster City, CA, USA) was used. Normalized promoter activity data are plotted in bar graphs representing mean ± SD from triplicate samples. Each independent experiment was repeated at least twice.

### MTS proliferation assay

Cells (1 × 10^3^ per well for NMuMG clones or 5 × 10^3^ per well for MDA-MB-231 clones) were seeded into a 96-well plate in replicates of 5, and cell proliferation was measured over 3 days using CellTiter 96 AQueous One Solution Cell Proliferation Assay (Promega Biotech AB, Nacka, Sweden) according to the manufacturer's instruction. Absorbance at 490 nm was read using a PerkinElmer EnSpire multimode reader (PerkinElmer, Waltham, MA, USA). The cell proliferation rate was calculated by dividing absorbance values over values read at day 0. All experiments were repeated at least twice.

### *In vitro* wound healing assay

Cells (1 × 10^6^ for NMuMG clones or 2 × 10^6^ for MDA-MB-231 clones) were seeded into a six-well plate and incubated for 24 h to achieve ∼80% confluency. A 1000-μl (for NMuMG clones) or 200-μl (for MDA-MB-231) micropipette tip was used to make three scratches in each well. For 5-aza-treated cells, 5-aza was added 2 days and replenished daily prior to scratching. Several regions were marked and photographed at 0, 8 or 24 h after the scratches were made. Phase-contrast microscopy images were taken using a Zeiss Axiovert 40 CFL microscope with a Zeiss Plan-neofluar 10×/0.3 objective lens and an AxioCam MRc digital camera. Image content was reduced and digital wound area measurements were taken using Adobe Photoshop CS3 Extended. The data are expressed as percentage of wound area that remained empty of cells at the time of measurement. Data are mean ± SD from nine independent measurements (microscopy fields). Each independent experiment was repeated at least twice.

### Invasion assay

To assess the cells’ invasive properties, 2.5 × 10^3^ cells (NMuMG clones) or 5 × 10^3^ cells (MDA-MB-231 clones) per 24-well were seeded into BioCoat GFR Matrigel invasion chambers (BD Biosciences, Stockholm, Sweden) with serum-free medium, according to the manufacturer's instructions. The transwells were embedded into complete medium and cells were allowed to invade for 24 h. At the end of the assay, the cells at the top (starting) side of the well were scraped and the cells (invading) on the bottom side of the well were photographed and counted. The data are expressed as percent of cells that invaded relative to the starting number of seeded cells. Data are mean ± SD from three independent measurements.

### Statistical analysis

Statistical analyses were performed by two-tailed unpaired Student's *t*-test. Significance was considered at *P* < 0.05 (*), *P* < 0.01 (**) or *P* < 0.001 (***).

## RESULTS

### Ectopic expression of HMGA2 leads to silencing of the *Cdh1* gene

**Figure 1.**
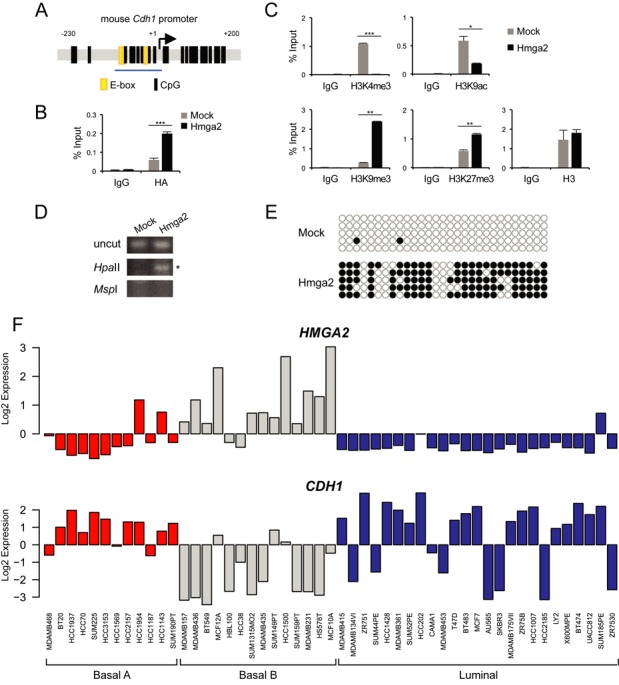
The *Cdh1* promoter is epigenetically silenced in HMGA2-overexpressing NMuMG cells. (**A**) An illustration of the mouse *Cdh1* promoter, position −230 to +200 base pairs relative to the transcription start site (+1), containing E-boxes (yellow) and CpG dinucleotides (black lines). The blue underline indicates the proximal region (position −108 to +3) examined in ChIP-qPCR assays in this study. (**B**) HMGA2 binding to proximal region of the mouse *Cdh1* promoter was analysed by ChIP assays with non-specific IgG or HA antibody in NM-Mock and NM-Hmga2 cells. Precipitated DNA was analysed by qPCR and data are graphed as explained in the methods. (**C**) ChIP-qPCR assays were performed to examine levels of histone H3 and its lysine modifications (active marks, K4me3 and K9ac; repressive marks, K9me3 and K27me3) on the proximal region of the mouse *Cdh1* promoter in NM-Mock and NM-Hmga2 cells. (**D**) HpaII–MspI digestion–methylation assay using primers which span the proximal region of the *Cdh1* promoter in NM-Mock and NM-Hmga2 cells. The PCR product was subjected to agarose gel electrophoresis and a band observed after HpaII-digestion indicates that the amplified DNA is methylated (asterisk). (**E**) The DNA methylation status of the *Cdh1* promoter in NM-Mock and NM-Hmga2 cells was analysed by bisulphite sequencing of the promoter region shown in panel A, where CpG sites are denoted by circles, and five independent clones of each cell line are shown here. White and black circles represent unmethylated and methylated CpG sites respectively. (**F**) Expression of *HMGA2* and *CDH1* in human breast cancer cell lines classified as basal A (red), basal B (grey) and luminal (blue) subtypes. Expression values derived from microarray analysis of gene expression are shown in logarithmic (log_2_) scale.

Alterations of the epigenome occur during cancer progression and the EMT process (24,25). In order to analyse the impact of HMGA2 on epigenetic regulation of the *Cdh1* promoter (Figure 1A), we performed ChIP assays to map histone modifications in the proximal region of the *Cdh1* promoter in epithelial NMuMG (NM-mock) cells or mesenchymal NM-Hmga2 cells which expressed high levels of EMT-TFs, like Snail and Twist (17,18). We chose to target the proximal *Cdh1* promoter region that is well studied in terms of its transcriptional and epigenetic regulation (6), and in addition, it spans across the well-conserved E-boxes (Figure 1A), where Snail binds and represses the *Cdh1* gene (26).

We first asked whether HMGA2 associates with this promoter region by using an HA antibody to immunoprecipitate HMGA2 as the protein has an HA-tag in NM-Hmga2 cells (12), and observed a strong binding of HMGA2 to the proximal *Cdh1* promoter in NM-Hmga2 cells relative to the significantly weaker binding obtained in NM-mock cells (Figure 1B). Tri-methylation of lysine 4 (H3K4me3) and acetylation of lysine 9 (H3K9ac) residues in histone H3 are associated with gene transcription, whereas tri-methylation of lysine 9 (H3K9me3) and lysine 27 (H3K27me3) residues are markers of gene repression (7). No H3K4me3 and low H3K9ac binding was observed in NM-Hmga2 cells (Figure 1C), which correlated with the lack of E-cadherin expression (Supplementary Figure S1A) (18). Increased occupancy of H3K9me3 or H3K27me3 in the *Cdh1* proximal promoter was found in NM-Hmga2 cells, as compared to NM-mock. In contrast, NM-mock cells expressed high levels of E-cadherin (Supplementary Figure S1A), and had higher content of H3K4me3 and H3K9ac, and lower content of H3K9me3 and H3K27me3 in the promoter (Figure 1C). We examined total histone H3 ChIP and saw that there was a similar degree of H3 occupancy at the proximal region in both cell types (Figure 1C).

NM-Hmga2 cells have high levels of Snail and Twist (Supplementary Figure S1A) which would contribute towards E-cadherin repression. Previously, we established NM-Hmga2 derivative clones with depletion of Snail, or depletion of Snail and Twist (18); these cells failed to re-express E-cadherin (Supplementary Figure S1A), despite the re-appearance of epithelial tight-junction markers, such as ZO-1 (18). We extended our ChIP analyses of histone modifications to the NM-Hmga2-shSnail or -shSnail-shTwist clones, and found that occupancy of H3K4me3 was very low and similar to background in NM-Hmga2 cells and NM-Hmga2-shSnail or -shSnail-shTwist clones (Supplementary Figure S1B). However, a gradual decrease of repressive H3K9me3 mark was observed when Snail was knocked down, which became much more significant when both Snail and Twist were knocked down in NM-Hmga2 cells (Supplementary Figure S1B), demonstrating that indeed Snail and Twist do contribute to E-cadherin repression, but in the presence of high HMGA2 levels, additional factors participate in the epigenetic silencing of *Cdh1*. As an additional specificity control, we stably silenced the overexpressed HMGA2 in NM-Hmga2 cells and examined the expression levels of E-cadherin (Supplementary Figure S1E). The efficiency of HMGA2 knockdown was strong (75% or higher), and all stable clones analysed demonstrated a relative expression of E-cadherin protein, albeit at low levels (Supplementary Figure S1E). Stable clones with the maximal knockdown of HMGA2 (e.g. clone nos. 73 and 86) showed the stronger expression of E-cadherin (Supplementary Figure S1E), suggesting that for efficient reversion of E-cadherin a complete lack of HMGA2 expression was necessary.

### HMGA2 enforces methylation on *Cdh1* CpG sequences

As DNA methylation of the *Cdh1* gene is also associated with its transcriptional repression (6), we proceeded to investigate the DNA methylation status of the *Cdh1* promoter in cells where HMGA2 was in abundance. Genomic DNA was isolated from NM-mock and NM-Hmga2 cells and digested with HpaII or MspI restriction enzymes. HpaII is methylation-sensitive and unable to cleave 5′-CCGG-3′ should the internal cytosine be methylated, whereas MspI is methylation-insensitive. Thus, a PCR product amplified after HpaII-digestion would reflect DNA methylation of a 5′-CCGG-3′ site. We used a set of primers which flanked the proximal E-boxes of the *Cdh1* gene (Figure 1A), as this region is part of a CpG island (www.genome.ucsc.edu). We could amplify a DNA band in the HpaII-digest of DNA from NM-Hmga2, but not in the HpaII-digested DNA from NM-mock cells, which indicates that the proximal *Cdh1* promoter was methylated following HMGA2 expression (Figure 1D). Similarly, in NM-Hmga2-shSnail and NM-Hmga2-shSnail-shTwist clones, the proximal regions were also methylated (Supplementary Figure S1C).

We confirmed the restriction enzyme-based promoter methylation results with sequence-specific analysis after modification of DNA by sodium bisulphite (Figure 1E). Indeed, essentially all CpG sequences of the proximal *Cdh1* promoter were devoid of methylation in NM-mock cells, whereas most of the CpG sequences were methylated in NM-Hmga2 cells (Figure 1E). Additionally, knockdown of Snail or Snail plus Twist left the methylation pattern of the *Cdh1* promoter essentially similar to the pattern of NM-Hmga2 cells (Supplementary Figure S1D). These data showed that HMGA2 was able to epigenetically repress the *Cdh1* gene, as shown by the gain of H3K9me3 and H3K27me3 marks and the DNA methylation in the proximal region of the *Cdh1* promoter. Furthermore, the repressive histone marks weakly changed whereas the repressive DNA methylation marks hardly changed when Snail, or both Snail and Twist, were knocked down in NM-Hmga2 cells, which agrees with the continuous loss of E-cadherin expression in these cells (Supplementary Figure S1A), and suggests that Snail and Twist are involved in the establishment of repressive histone marks and DNA methylation, whereas long-term chromatin repression and DNA methylation may depend on the continuously high HMGA2 input.

Considering that HMGA2 is a chromatin factor, the NM-Hmga2 cells provide an ectopic mouse cell model with possible genomic instability that may attribute to the EMT and invasive capacity (see invasion in the ‘Results’ section) of breast cells. We therefore compared the expression patterns of *HMGA2* and *CDH1* in human breast cancer cells using the GOBO tool (21). High expression of *HMGA2* and corresponding low expression of *CDH1* were found in cell lines of the basal B subtype, compared to the basal A or luminal subtypes (Figure 1F). Classification of the subtypes were based on Neve *et al.* (27); cells classified as basal B subtype were described to be more mesenchymal and highly invasive, while luminal epithelial cells were more differentiated and less invasive (27), suggesting that HMGA2 and E-cadherin play opposite roles in EMT and invasiveness.

### 5-Aza-2′-deoxycytidine counteracts HMGA2-mediated repression of *Cdh1* and blocks cell migration

**Figure 2.**
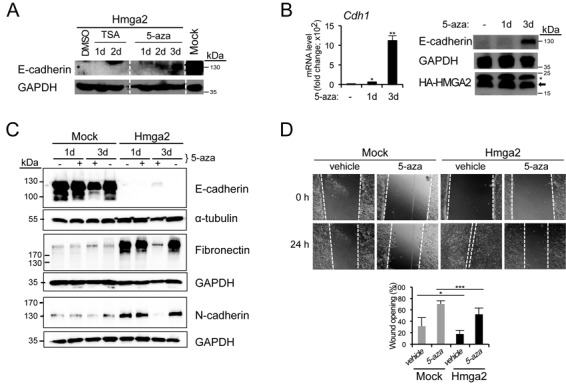
5-Aza-2'-deoxycytidine treatment restores E-cadherin expression. (**A**) Immunoblots for E-cadherin and GAPDH (loading control) of NM-Hmga2 cells treated with vehicle (DMSO), TSA (100 ng/ml) or 5-aza (20 μM) for 1, 2 or 3 days, and untreated NM-Mock cells. (**B**) E-cadherin mRNA and protein levels in NM-Hmga2 cells treated with vehicle (−) or 5-aza for 1 or 3 days. Immunoblots for E-cadherin, HMGA2 (arrow) and GAPDH are included. Asterisk indicates unspecific bands. (**C**) Immunoblots for E-cadherin, fibronectin and N-cadherin of NM-Mock and NM-Hmga2 cells treated with vehicle or 5-aza (20 μM) for 1 or 3 days (+) and untreated cells for the same period of time (−). GAPDH and α-tubulin serve as loading controls. (**D**) Wound healing assay of NM-Mock and NM-Hmga2 cells treated with vehicle or 5-aza. 5-Aza was added 24 h before the scratch was made (0 h) and measurements were taken 24 h after the wounding. 5-Aza was replenished every day and cells were cultured in the presence of 5-aza or vehicle for total of 3 days. The bar graph shows wound area at 24 h as a percentage of original wound area at 0 h (right panel; mean ± SD from nine fields).

In order to test the functional relevance of repressive histone marks and DNA methylation on the *Cdh1* promoter established by HMGA2, we treated NM-Hmga2 cells with an HDAC inhibitor, trichostatin A (TSA), or with the DNA demethylating drug, 5-aza-2′-deoxycytidine (5-aza), over the course of a few days to see if the *Cdh1* gene could be reactivated. Snail can repress E-cadherin by the recruitment of HDACs (26) and Snail expression is high in NM-Hmga2 cells, however, treatment of cells with the HDAC inhibitor TSA was not sufficient to de-repress E-cadherin over a period of 2 days (Figure 2A); instead, it induced cell death after 3 days of exposure, resulting in the loss of sample (not shown). E-cadherin re-expression at mRNA and protein level was however observed after a 3-day treatment at a high concentration of 20 μM 5-aza (Figure 2A and B). It must be noted that the 5-aza-treated cells appeared somewhat unhealthy, which was expected based on the established cytotoxic effects of 5-aza (28). Treatment with lower 5-aza concentrations was not effective (data not shown). Importantly, under these conditions, the cells continued to express high levels of HMGA2 (Figure 2B). Analysis of two mesenchymal markers, fibronectin and N-cadherin, which are potently expressed in NM-Hmga2 cells, showed that the 3-day treatment with 5-aza significantly suppressed expression of these two proteins (Figure 2C), which agreed with the change of morphology in the treated cells (data not shown). The data suggest that E-cadherin inactivation in NM-Hmga2 cells is dependent on the action of DNA methylating enzymes and strong demethylating agents such as 5-aza can induce a global reversion towards the epithelial phenotype, despite the high expression level of HMGA2.

Given that E-cadherin is an established suppressor of invasion (29,30) and that HMGA2 elicits EMT (12), we hypothesized that HMGA2 could have a role in cell migration and invasion. In a classical *in vitro* wound-healing assay, NM-Hmga2 cells were able to migrate and re-populate the wound area after 8 h, and completely (>80%) sealed the scratch after 24 h, whereas NM-mock cells only sealed 60% of the area after 24 h (Supplementary Figure S2A). In addition, the proliferation rates of NM-mock and NM-Hmga2 cells were similar up to 3 days (Supplementary Figure S2B), indicating that the differences observed in the wound closure between the cell models were mainly due to cell migration and not proliferation. We also assessed the invasive ability of these cells using a Matrigel transwell assay; NM-Hmga2 cells were found to be significantly more invasive compared to NM-mock cells (Supplementary Figure S2C). As predicted from the impact of the demethylating agent 5-aza on E-cadherin expression, treatment of the mammary epithelial or mesenchymal cells with 5-aza led to a significant degree of inhibition of cell migration during the course of 24 h, in both NM-mock (epithelial) and NM-Hmga2 (mesenchymal) cells (Figure 2D). This suggests that mechanisms that target DNA methylation have an impact on the migratory capacity of breast cancer cells. In summary, all above data suggest that DNA methylation is a major mechanism for the long-term silencing of the *Cdh1* gene when HMGA2 is overexpressed.

### HMGA2 controls E-cadherin expression, migration and invasion in human breast cancer cells

Stimulated by the above results obtained from transfected NMuMG cells, we switched to a well-studied human breast cancer cell line MDA-MB-231 since, (i) these cells are highly invasive and metastatic and are characterized by a mesenchymal phenotype (20); (ii) they do not express E-cadherin and (iii*)* they exhibit extensive *CDH1* promoter hypermethylation (8). In addition, and relevant to this study, the MDA-MB-231 cell line also expressed high levels of endogenous HMGA2, compared to many epithelial breast cancer cell lines of the luminal type, such as MCF7, which expressed E-cadherin but low amounts of HMGA2 (Figure 1F). We transiently knocked down HMGA2 in MDA-MB-231 cells with two individual siRNAs, no. 1 and no. 4, or a mix of both, and then performed a wound healing assay. After 24 h, the scratch in the control (si*Luc*) cells was almost sealed, whereas silencing of HMGA2 slowed down cell migration (Supplementary Figure S3A), despite the presence of single invasive cells in the wound area. The siRNA knockdown efficiency of HMGA2 was also verified as >85% (Supplementary Figure S3B).

**Figure 3.**
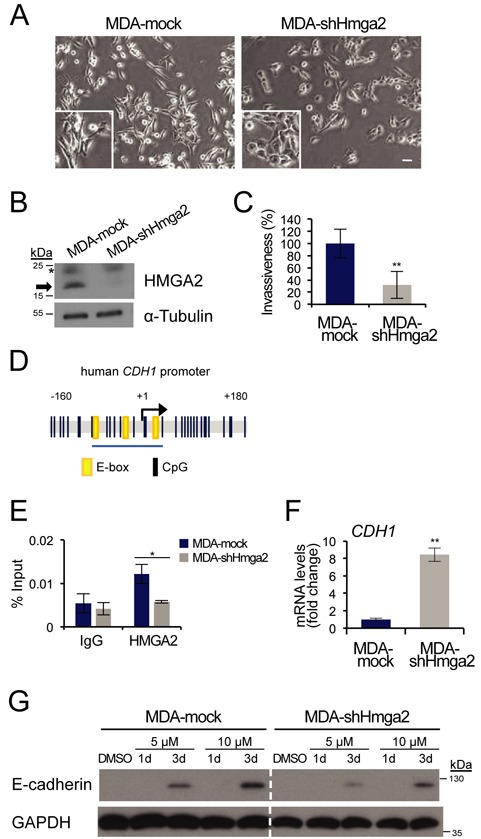
Depletion of HMGA2 leads to increase of E-cadherin and reduced invasion. (**A**) Cellular morphology of MDA-MB-231 stable clones with HMGA2 knocked down (MDA-shHmga2) or empty pRetroSuper vector (MDA-mock). *Scale bar*: 50 μm. (**B**) Immunoblot analysis of endogenous HMGA2 in MDA-mock and MDA-shHmga2 cells (arrow) and α-tubulin serves as loading control. Asterisk indicates unspecific bands. (**C**) Invasion ability of MDA-mock and MDA-shHmga2 cells was assessed using a Matrigel transwell assay. Bar graph shows invasion rate relative to MDA-mock cells, which was set at 100% (mean ± SD values from triplicates). (**D**) An illustration of the human *Cdh1* promoter, position −160 to +180 base pairs relative to the transcription start site (+1), containing E-boxes (yellow) and CpG dinucleotides (black lines). The blue underline indicates the proximal region (position −73 to +26) examined in ChIP-qPCR assays. (**E**) Human *CDH1* promoter binding by endogenous HMGA2 was analysed by ChIP-qPCR in MDA-mock and MDA-shHmga2 cells. (**F**) E-cadherin (*CDH1*) mRNA analysis in MDA-mock and MDA-shHmga2 cells, normalized to *GAPDH* mRNA expression. Each bar represents mean ± SD values from triplicate samples and *CDH1* levels in MDA-mock cell are normalized to 1. (**G**) MDA-mock and MDA-shHmga2 cells treated with vehicle or the indicated concentrations of 5-aza for 1 or 3 days, and immunoblotted for E-cadherin and GAPDH (loading control).

We went on to establish stable clones with HMGA2 knockdown in MDA-MB-231 cells using shRNA vectors (MDA-shHmga2). The efficiency of HMGA2 knockdown was between 50 and 80% in four clones as determined by RT-qPCR (data not shown); one of the four clones (clone no. 4, here referred as MDA-shHmga2) and one vector control clone (MDA-mock) were selected for further characterization (Figure 3A and B).

Morphological analysis of MDA-mock cells displayed similar phenotype as their parental MDA-MB-231 cells: singly dispersed cells with spindle-like protrusions and mesenchymal features (Figure 3A). In contrast, MDA-shHmga2 cells lost their spindle-like morphology, exhibiting reduced protrusive ends and formed cell islets, an indication of a gain in cell–cell adhesion (Figure 3A). This phenotypic change could possibly be explained by a regulatory effect of HMGA2 on E-cadherin, similar to the effect established in the NM-Hmga2 cell model. We also observed that MDA-shHmga2 cells grew slightly slower than MDA-mock cells (Supplementary Figure S3C), which could be possibly explained by the postulated role of HMGA2 in regulating expression of cyclins (31). More dramatic was the effect of HMGA2 silencing on invasion of MDA-MB-231 cells through Matrigel, which led to a higher than 2-fold block in invasive capacity (Figure 3C). Possible mediators of HMGA2′s impact on invasion could be mesenchymal genes, e.g. fibronectin, vimentin and N-cadherin, which are known to be regulated by HMGA2 (18). However, there was no change in fibronectin, vimentin and N-cadherin protein levels in MDA-shHmga2 cells when compared to the MDA-mock cells (data not shown), while significant upregulation of the pro-epithelial transcription factor KLF4 (Krüppel-like factor 4) was observed after stable HMGA2 knockdown (Supplementary Figure S3D). On the other hand, additional mRNA analyses of selected matrix remodelling proteins known to have a role in invasion and metastasis (3,32), revealed that the pro-invasive genes *MMP2* (matrix metalloprotease 2) and *TNC* (tenascin C) were downregulated in MDA-shHmga2 cells, but to our surprise, *MMP1* was upregulated (Supplementary Figure S3D). There were no changes in mRNA levels for *MMP9* and *MMP10* genes (data not shown). On the other hand, the antagonists of MMPs, encoded by the *TIMP1* and *TIMP3* genes, were found to be upregulated (Supplementary Figure S3D). Taken together, HMGA2 seems to confer cancer cells with migratory and invasive properties, which links well with the pro-mesenchymal and pro-invasive activities HMGA2 elicits in mammary epithelial cells (Figure 2). The pro-invasive properties of HMGA2 involve first the efficient downregulation of E-cadherin (Supplementary Figure S1A, (12)), and additionally the possible involvement of matrix remodelling and pro-invasive proteins, whose levels are regulated by HMGA2 (Supplementary Figure S3D).

The *CDH1* promoter region is highly conserved between mouse and human, except for an E-box that is present after the TSS in the human sequence (Figure 3D) (6). We designed a set of primers homologous to the mouse *Cdh1* promoter that overlapped the E-boxes and part of a CpG island, and performed ChIP assays with these primers in MDA-mock or MDA-shHmga2 cells. ChIP analysis first demonstrated that endogenous HMGA2 occupied the proximal promoter of the human *CDH1* gene in MDA-mock cells, whereas the association of HMGA2 was decreased to the level of background non-specific IgG control in MDA-shHmga2 cells (Figure 3E). The chromatin recruitment profile of HMGA2 correlated strongly to the expression level of endogenous *E-cadherin* mRNA, as the E-cadherin transcript was significantly upregulated in MDA-shHmga2 cells (Figure 3F). However, we were not able to correlate these mRNA levels to a corresponding increase at protein expression (Figure 3G, sixth lane, DMSO control). This apparent lack of E-cadherin protein expression in the MDA-shHmga2 stable clone suggested that the *CDH1* promoter might still be hypermethylated. Indeed, treatment of MDA-shHmga2 cells with 5 μM 5-aza for 3 days, effectively restored E-cadherin expression; this effect was further enhanced by 10 μM 5-aza (Figure 3G). The same was observed for the control MDA-mock clone (Figure 3G). These observations suggest that knockdown of HMGA2 effectively derepresses the *CDH1* gene so that an 8-fold induction of *CDH1* mRNA could be observed (Figure 3F), however, in order to observe an effect on E-cadherin protein levels, an even higher derepression is necessary, which was achieved via the action of 5-aza (Figure 3G). Interestingly, 5-aza treatment significantly blocked breast cancer cell migration in both MDA-mock and MDA-shHmga2 cells (Supplementary Figure S3E). Since E-cadherin protein levels had not recovered under these conditions (10 μM 5-aza for 24 h, Figure 3G), the impact of 5-aza on cell migration may possibly reflect perturbation at the level of MMPs and TIMPs, which is suggested by the expression profile of MDA-shHMGA2 cells (Supplementary Figure S3E).

### Impact of TGFβ on E-cadherin repression and DNMT3A induction

**Figure 4.**
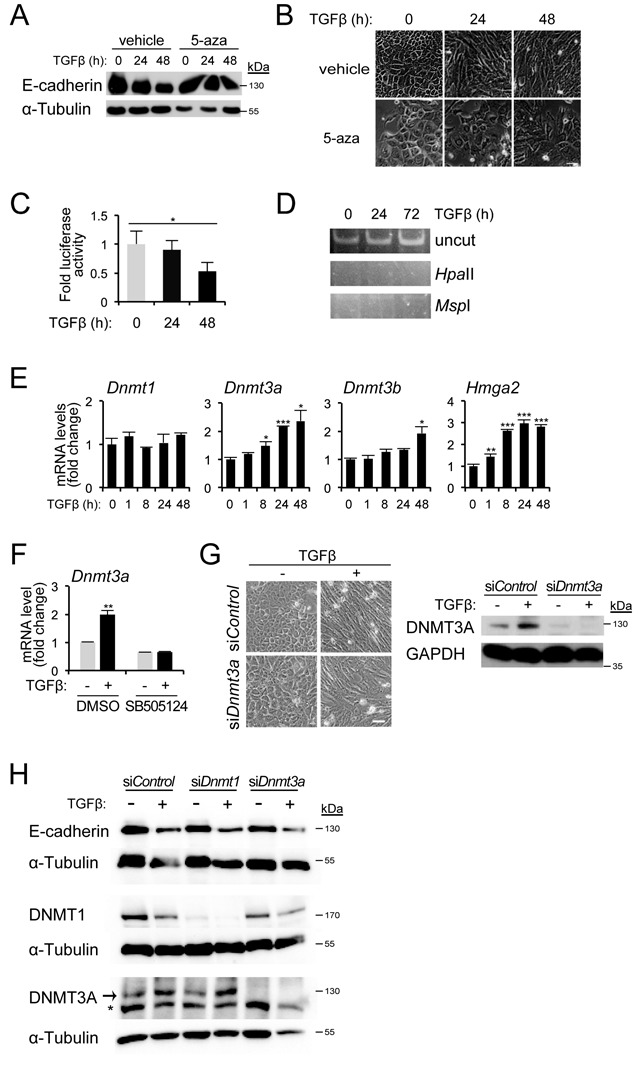
DNMT3A upregulation by TGFβ in NMuMG cells. (**A**) Immunoblots for E-cadherin and GAPDH in NMuMG cells unstimulated or stimulated with TGFβ1 (5 ng/ml) for 24 or 48 h, in the presence of vehicle or 10 μM 5-aza. (**B**) Phase contrast microscopy of cells as described in (A). *Scale bar:* 50 μm. (**C**) Luciferase reporter assay of human *Cdh1* promoter in NMuMG cells stimulated with TGFβ1 for the indicated time period. (**D**) HpaII–MspI digestion–methylation assay of *Cdh1* promoter in NMuMG cells stimulated with TGFβ1 for the indicated time period. (**E**) *Dnmt* mRNA levels after TGFβ1 treatment of NMuMG cells for the indicated time periods, were normalized to *Gapdh* mRNA expression. Expression values of the 0-h time point are normalized to 1. (**F**) *Dnmt3a* mRNA expression in NMuMG cells pre-treated for 30 min with or without TGFβ type I receptor kinase inhibitor SB505124 (10 μM), in the absence or presence of TGFβ1 for 24 h. DMSO was used as a vehicle. qPCR values were normalized to that of *Gapdh* and the expression values of DMSO-treated, unstimulated cells were normalized to 1. (**G**) Phase contrast images (left panel) and immunoblot analysis (right panel) of DNMT3A and GAPDH protein levels in NMuMG cells transfected with si*Control* or si*Dnmt3a*, untreated or treated with TGFβ1 for 24 h. *Scale bar*: 50 μm. (**H**) E-cadherin, DNMT1, DNMT3A and α-tubulin protein levels in NMuMG cells transfected with si*Control*, si*Dnmt1* or si*Dnmt3a*, in the absence or presence of TGFβ1 for 48 h.

All previous experiments were performed in NMuMG or MDA-MB-231 cells using genetic perturbation of HMGA2 expression (overexpression or silencing mediated by siRNAs or shRNAs). In order to confirm our findings in a more physiological setting where endogenous HMGA2 levels can be regulated, we employed parental NMuMG cells that undergo robust EMT in response to TGFβ, including downregulation of E-cadherin (12). Treatment of NMuMG cells with TGFβ1 led to a time-dependent E-cadherin downregulation, which was blocked after simultaneous treatment of the cells with 5-aza (Figure 4A). This effect correlated to the overall morphology of the cell cultures, whereby TGFβ1 induced cell–cell detachment and elongated, mesenchymal cell morphology, while 5-aza together with TGFβ1 blocked the elongated, mesenchymal phenotype and resulted in more cuboidal and compact cell islands (Figure 4B). Under the same conditions, transient transfection of a human *CDH1* proximal promoter-luciferase construct (22) into NMuMG cells, led to a time-dependent downregulation of luciferase output when the cells were stimulated with TGFβ1 (Figure 4C). DNA methylation analysis of the endogenous mouse *Cdh1* proximal promoter using the HpaII/MspI restriction enzyme assay failed to demonstrate efficient methylation of the promoter during the first 24–72 h of stimulation with TGFβ1 (Figure 4D). This result suggested that the epigenetic effect obtained after long-term and stable expression of HMGA2 on the *Cdh1* promoter is much stronger than that employed by transient stimulation of EMT by TGFβ.

In addition, we analysed the expression levels of the three endogenous DNMTs in NMuMG cells responding to TGFβ for as long as 48 h (Figure 4E). While *Dnmt1* mRNA levels did not change during the TGFβ1 time-course, *Dnmt3a* mRNA levels were significantly upregulated after 8 h and reached a plateau during the 24–48 h interval, whereas *Dnmt3b* mRNA levels showed significant upregulation only with a very late onset after 24 h (Figure 4E). Analysis of endogenous *Hmga2* mRNA induction by TGFβ1 during the same time-course experiment confirmed our previous findings (12), and demonstrated that Hmga2 upregulation began as early as 1 h after TGFβ1 stimulation, and reached a plateau after 8 h and remained high at a sustained level over the 48 h period (Figure 4E). The data suggest that DNMT3A, and to a lesser extent DNMT3B, are late targets of the TGFβ signalling pathway and possibly their regulation lies downstream of HMGA2.

Based on the kinetic profile that showed more robust regulation of *Dnmt3a* mRNA by TGFβ (Figure 4E), we focused on the functional analysis of this DNA methyltransferase. The induction of *Dnmt3a* mRNA by TGFβ1 could be inhibited by the specific TGFβ type I receptor kinase inhibitor SB505124; even the basal levels without exogenous TGFβ1 stimulation were suppressed (Figure 4F). Moreover, cells transfected with *Dnmt3a* siRNA underwent mesenchymal cell elongation and cell–cell detachment when treated with TGFβ1, even though the morphology of these cells was less elongated compared to the siControl-transfected cells treated with TGFβ (Figure 4G). Stimulation of parental NMuMG cells with 5 ng/ml TGFβ1 for 24 h after control siRNA transfection led to an upregulation of DNMT3A protein level (Figure 4G). As expected, the upregulation and basal endogenous DNMT3A protein expression was blocked when we knocked down DNMT3A by siRNA (Figure 4G). Further analysis of E-cadherin protein levels in NMuMG cells that were transiently transfected with siRNAs targeting DNMT1 and DNMT3a revealed that E-cadherin downregulation induced by a 48 h stimulation with TGFβ1 could not be blocked by either siRNA (Figure 4H). These experiments also revealed a downregulation of DNMT1 protein by TGFβ (Figure 4H), which did not correlate with the corresponding mRNA levels of *Dnmt1* (Figure 4E), and thus suggested a possible post-transcriptional effect of TGFβ on DNMT1, which we have not yet analysed deeper. All the above data suggest that DNMT3a might be a candidate epigenetic regulator acting downstream of HMGA2, however, during the first period of TGFβ signalling in NMuMG cells, this DNA methyltransferase does not seem to contribute to the onset of EMT and E-cadherin downregulation. At this early stage of the EMT program, TGFβ and HMGA2 operate at the level of Snail recruitment to target genes, such as E-cadherin, affecting local histone modifications and preparing the genomic landscape for subsequent irreversible DNA methylation including the *E-cadherin* locus.

### Epigenetic control on the *Cdh1* promoter during TGFβ-induced EMT

**Figure 5.**
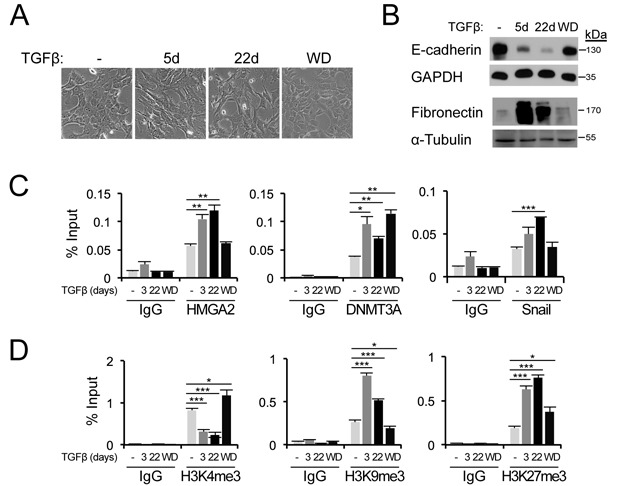
TGFβ-induced EMT is associated with epigenetic changes on the *Cdh1* promoter. (**A**) Morphology of parental NMuMG cells untreated, treated with TGFβ1 (1 ng/ml) for the indicated time periods, or treated with TGFβ1 for 22 days with subsequent withdrawal of TGFβ1 for another 14 days (WD). *Scale bar*: 50 μm. (**B**) Immunoblot analyses for E-cadherin and fibronectin protein levels in cells as described in (A). GAPDH and α-tubulin serve as loading control. (**C**) Binding of endogenous HMGA2, Snail and DNMT3A on the mouse *Cdh1* promoter analysed by ChIP-qPCR in cells described in (A). (**D**) ChIP-qPCR analyses of active H3K4me3 marks and repressive H3K9me3 and H3K27me3 marks at the *Cdh1* promoter in cells described in (A).

In order to address the above suggestive conclusion, we stimulated NMuMG cells with TGFβ1 for 5 and 22 days; after 22 days we removed TGFβ1 from the cell medium (Figure 5A) in order to enforce reversion of the cell phenotype to an epithelial as we have previously established (33). Robust and sustained mesenchymal cell morphology could be observed during the course of 5–22 days and reversion to an epithelial morphology of more compact cell islands was evident 2 weeks after TGFβ1 withdrawal (Figure 5A). The morphological changes observed under the microscope were nicely corroborated at the molecular level by examining E-cadherin (epithelial) and fibronectin (mesenchymal) protein regulation (Figure 5B). Time-dependent E-cadherin downregulation and fibronectin upregulation were both completely reverted to the state of control unstimulated NMuMG cells 14 days after TGFβ1 withdrawal (Figure 5B). Using these conditions of EMT and epithelial reversion, we analysed time-dependent recruitment of endogenous HMGA2, Snail and DNMT3A proteins to the proximal *Cdh1* promoter using ChIP analysis (Figure 5C), and correlated their pattern of recruitment to the pattern of histone modification on the promoter (Figure 5D). Endogenous HMGA2 was recruited to the proximal *Cdh1* promoter in a time dependent manner, and this recruitment was sustained for up to 22 days of TGFβ1 stimulation, whereas an additional 14 days of TGFβ1 withdrawal returned the HMGA2 binding profile to the control level of epithelial unstimulated NMuMG cells (Figure 5C). The same pattern was observed for endogenous Snail that showed sustained recruitment and complete reversion after withdrawal (Figure 5C). Unexpectedly, DNMT3A was also recruited to the promoter in a time-dependent manner; however, DNMT3A remained bound to the promoter in cells that had reverted to an epithelial phenotype and re-expressed E-cadherin after TGFβ1 withdrawal (Figure 5C). The latter finding suggests that additional mechanisms might operate to inactivate DNMT3A function despite its presence on the promoter, or that re-expression of E-cadherin is independent from the presence of DNMT3A on the promoter. In the same experimental set up, the active chromatin mark of histone H3K4me3 was dramatically reduced upon TGFβ stimulation and reverted back after its withdrawal, while the two repressive chromatin marks of histone H3K9me3 and H3K27me3 were strongly induced by TGFβ and reverted back to low levels after growth factor withdrawal (Figure 5D). These observations let us to conclude that during the dynamic process of EMT, promoted in NMuMG cells by long-term TGFβ stimulation, dynamic changes in transcription factor recruitment (HMGA2, Snail) and corresponding histone modifications, take place, while DNMT3A follows a more complicated pattern of recruitment to the *E-cadherin* gene promoter and DNA methylation probably requires more sustained and powerful activity by the EMT-TFs.

### HMGA2 and DNMT3A cooperation in mesenchymal breast cells

**Figure 6.**
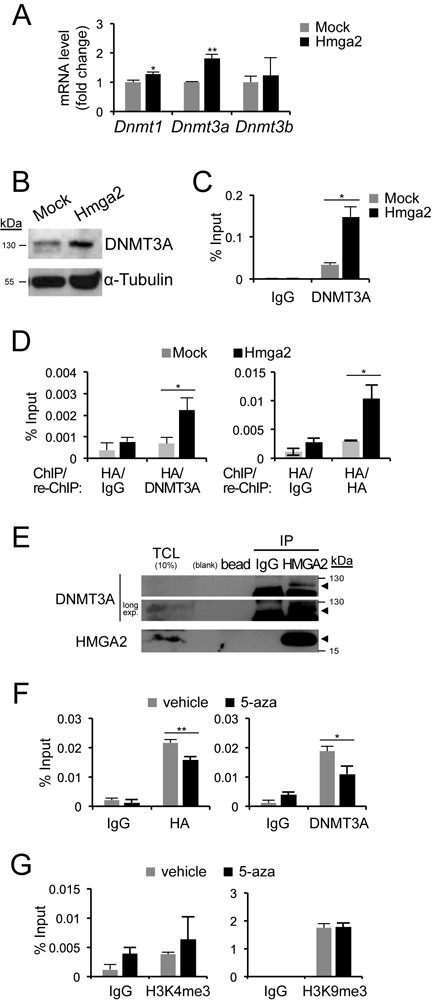
HMGA2 and DNMT3A bind to the *Cdh1* promoter. (**A**) *Dnmt* mRNA expression levels in NM-Mock and NM-Hmga2 cells were normalized to *Gapdh* mRNA expression and the expression values of NM-Mock cells were normalized to 1. (**B**) Immunoblot analyses of DNMT3A and α-tubulin protein levels in NM-Mock and NM-Hmga2 cells. (**C**) ChIP-qPCR analysis with IgG or DNMT3A antibody on *Cdh1* promoter in NM-Mock and NM-Hmga2 cells. (**D**) ChIP assays with HA antibody, followed by a sequential ChIP (re-ChIP) with IgG, HA or DNMT3A antibody were performed and analysed by qPCR on *Cdh1* promoter in NM-Mock and NM-Hmga2 cells. (**E**) Immunoprecipitation of endogenous HMGA2, followed by immunoblotting for endogenous DNMT3A in HEK293T cells. IP, immunoprecipitation; TCL, total cell lysate; long exp*.*, long exposure. Arrowheads mark specific protein bands. (**F**) ChIP-qPCR analyses of HA-HMGA2 and endogenous DNMT3A binding to the *Cdh1* promoter in NM-Hmga2 cells treated with vehicle (−) or 5-aza (20 μM) for 3 days. (**G**) ChIP-qPCR analyses of active histone mark H3K4me3 and repressive histone mark H3K9me3 levels in NM-Hmga2 as described in panel F.

As the *Cdh1* promoter DNA was hypermethylated in NM-Hmga2 cells (Figure 1D and E), we analysed mRNA levels of the DNMT family members (*Dnmt1*, *Dnmt3a* and *Dnmt3b*) in NM-mock and NM-Hmga2 cells (Figure 6A). Small differences in *Dnmt1* or *Dnmt3b* mRNA levels were recorded between the two stable clones; however, NM-Hmga2 cells expressed higher levels of Dnmt3a mRNA and protein (Figure 6A and B). Therefore, the profile of DNMT expression in mesenchymal NM-Hmga2 cells correlated with that in parental NMuMG cells stimulated with TGFβ (Figure 4E). In addition, and similar to NMuMG cells under TGFβ stimulation (Figure 5C), NM-Hmga2 cells exhibited significant levels of endogenous DNMT3A associated with the *Cdh1* promoter, whereas NM-Mock cells showed only weak binding of DNMT3A to the promoter (Figure 6C). Interestingly, the NM-Hmga2 cell clones where Snail or Snail plus Twist were stably knocked down continued to demonstrate significant association of DNMT3A to the *Cdh1* promoter (Supplementary Figure S1F).

Since both HMGA2 and DNMT3A associate with the same, relatively short *Cdh1* promoter region, we postulated that the proteins may interact and be recruited together on the promoter. To demonstrate association of the two proteins at the endogenous level and on the *Cdh1* promoter chromatin, a ChIP experiment for HMGA2 followed by re-ChIP for DNMT3A was performed in NM-Mock and NM-Hmga2 cells (Figure 6D). Only in NM-Hmga2 cells could we observe reproducible and significant association of the two proteins to the *Cdh1* promoter (Figure 6D). Compared to the ChIP-re-ChIP assay for HMGA2 only, which resulted in a 3-fold enrichment of HMGA2 to the *Cdh1* promoter in NM-Hmga2 cells relative to NM-Mock, the ChIP-re-ChIP assay for HMGA2 and DNMT3A demonstrated a similar 3-fold enrichment of the HMGA2/DNMT3A complex on the promoter (Figure 6D). This suggested that a significant proportion of HMGA2 bound to the *Cdh1* promoter was in complex with DNMT3A. In agreement with the data from mesenchymal NM-Hmga2 cells, the protein association could be verified using a co-immunoprecipitation assay, where endogenous HMGA2 pulled down endogenous DNMT3A in HEK293T cells that express detectable levels of HMGA2 due to their embryonic origin (Figure 6E).

We also tested the impact of the demethylating agent 5-aza on the recruitment of HMGA2 and DNMT3A on the *Cdh1* promoter (Figure 6F). To our surprise, we found that 5-aza caused a reduction in both HMGA2 and DNMT3A associations to the *Cdh1* promoter (Figure 6F), indicating that 5-aza affected their binding efficiencies to DNA at this region, thus alleviating *Cdh1* repression. 5-Aza is known to also affect histone modifications (34), which could provide an additional mechanism of reactivating the *Cdh1* promoter in NM-Hmga2 cells, other than direct DNA demethylation. We therefore compared the occupancies of H3K4me3 and H3K9me3 on the *Cdh1* promoter in 5-aza-treated NM-Hmga2 cells to untreated cells (Figure 6G). There were no significant differences in H3K4me3 and H3K9me3 binding after 5-aza treatment. We conclude that 5-aza did not have any major effects on the histone modifications we studied, but rather acted more directly on the binding of the HMGA2–DNMT3A protein complex.

### HMGA2 contacts and prohibits CTCF from binding efficiently to the *Cdh1* promoter

**Figure 7.**
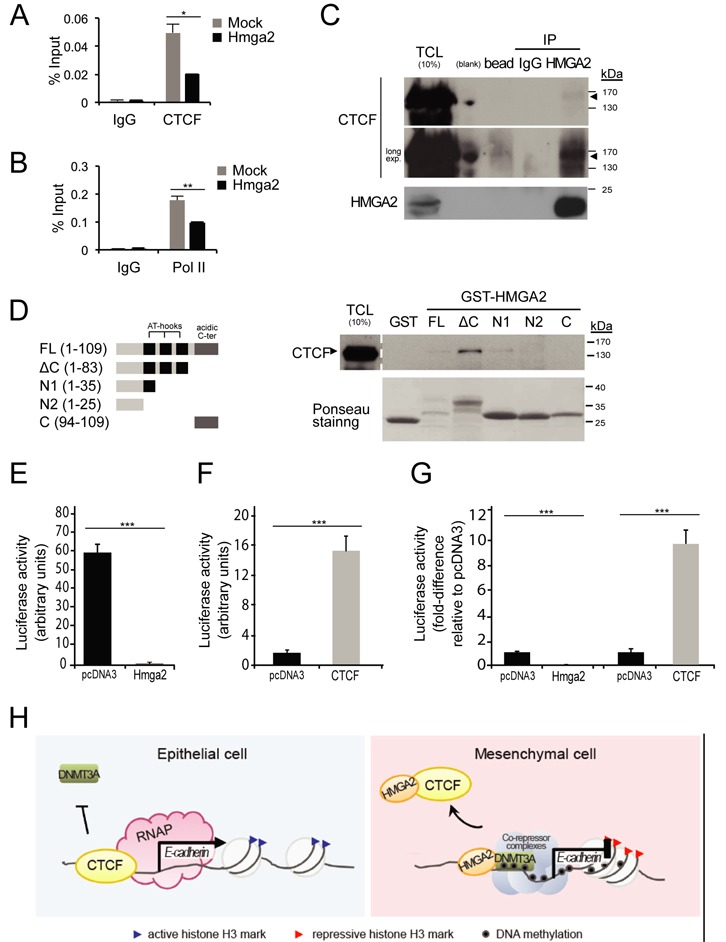
Impact of HMGA2 on CTCF binding to chromatin. (**A**, **B**) ChIP-qPCR analyses of CTCF and RNA polymerase (Pol II) on the *Cdh1* promoter in NM-Mock and NM-Hmga2 cells. (**C**) Immunoprecipitation of endogenous HMGA2, followed by immunoblotting for endogenous CTCF in HEK293T cells. IP, immunoprecipitation; TCL, total cell lysate; long exp., long exposure. Arrowheads mark specific protein bands. (**D**) Purified GST or GST-HMGA2 and its deletion mutants, as schematically depicted in the left panel, were used in a pull-down assay with HEK293T cell extracts and immunoblotted for endogenous CTCF. Input of GST fusion proteins used in the pull-down were visualized by Ponceau-S staining. FL, full-length; ΔC, deletion of C-terminal; N1, N-terminal 1; N2, N-terminal 2; C, C-terminal; TCL, total cell lysate. (**E**, **F**) Luciferase reporter assays of human *Cdh1* promoter in NMuMG cells co-transfected with empty vector pcDNA3 or pDNA3-HA-HMGA2 (E) or pcDNA3-Flag-CTCF (F). Each bar represents mean ± SD values from triplicate samples. ***P < 0.001, compared to empty vector-transfected cells. (**G**) The data of panels E and F are re-plotted on a single graph after normalization of the empty vector pcDNA3 conditions to 1, for better comparative visualization. (**H**) CTCF prevents the spread of heterochromatin and DNA methylation, and promotes E-cadherin transcription by RNA polymerase II complexes (RNAP) in epithelial cells. HMGA2 interacts with CTCF such that CTCF can no longer bind efficiently and protect the *Cdh1* region from repressive complexes, e.g. DNMT3A, which enforces methylation of DNA at the locus. Additional co-repressor complexes may be recruited to the locus. The key EMT-TFs, such as Snail, that initiate *Cdh1* repression, and which cooperate with HMGA2 are not shown for simplicity.

We additionally hypothesized that HMGA2, being a chromatin remodeler, could influence other factors that control chromatin dynamics. For example, HMGA2 might perturb the chromatin boundaries insulated by the CCCTC-binding factor (CTCF) on the *Cdh1* promoter. CTCF insulates and shields tumour suppressor genes, like *Cdh1*, *p16* and *RASSF1A*, from being epigenetically silenced in cancers (35); on the other hand, when such DNA loci become methylated, CTCF gets repelled or is unable to bind anymore (36). In NM-Hmga2 cells where HMGA2 expression is high, we found a loss of binding by CTCF to the *Cdh1* promoter, compared to NM-mock cells (Figure 7A). This correlated with reduced occupancy by RNA polymerase II (Pol II) at the same promoter region (Figure 7B). These results are compatible with the established mechanisms that prohibit CTCF from binding to DNA methylated regions, and the Pol II ChIP results correlate with the observed silencing of the *Cdh1* gene when HMGA2 is overexpressed. In addition, the inverse correlations were observed when HMGA2 was knocked down in MDA-MB-231 breast cancer cells (Supplementary Figure S4A (i,ii)); CTCF and Pol II recruitment to the *CDH1* promoter were correspondingly enhanced.

The ChIP analysis of CTCF suggested that HMGA2 and CTCF might form complexes, and possibly, that such a complex interferes with the binding of CTCF to the *Cdh1* promoter. Co-immunoprecipitation of the endogenous proteins in embryonic HEK293T cells revealed the formation of a protein complex between HMGA2 and CTCF (Figure 7C). The efficiency of this co-precipitation was weak but reproducibly higher when compared to a nonspecific IgG control or affinity beads alone (Figure 7C). Using a panel of HMGA2 protein deletions expressed as recombinant GST-fusion proteins in *Escherichia coli* demonstrated again the weak association of full-length HMGA2 or its N-terminal domain including the first AT-hook with CTCF, while deletion of the C-terminal regulatory domain of HMGA2 dramatically enhanced the association with CTCF (Figure 7D). The isolated N- and C-terminal domains of HMGA2 completely failed to show protein interactions with CTCF (Figure 7D). The same results were obtained using cell extracts from two independent breast cancer cell models, MDA-MB-231 and MCF10CA1a (Supplementary Figure S4B). In fact, in these two cell models binding of endogenous CTCF to the full-length HMGA2 was readily observed, in addition to the binding of the deletion mutants that lack the C-terminal domain (ΔC) or include the N-terminal domain with the first AT-hook (N1) (Supplementary Figure S4B). Using the same human E-cadherin promoter fragment analyzed above under TGFβ stimulation conditions (Figure 4C), we tested the impact of exogenous HMGA2 and CTCF on promoter activity (Figure 7E–G). In transiently transfected NMuMG cells, the potent activity of the *CDH1* proximal promoter-luciferase construct was dramatically repressed by HMGA2 (Figure 7E). In contrast, overexpression of CTCF further super-activated the *CDH1* proximal promoter-luciferase construct (Figure 7F). When we co-expressed HMGA2 and CTCF by titrating up each expression vector, we could observe antagonistic action of HMGA2 over CTCF as expected, however we failed at generating results of strong statistical significance (data not shown). Figure 7G shows the same data as Figure 7E and F when the basal promoter activities are normalized to 1 in the transfected NMuMG cells, and emphasizes the opposing actions of HMGA2 and CTCF on the cloned *CDH1* promoter fragment.

In the breast cancer MDA-MB-231 cell model, histone H3 K4me3 and K27me3 modifications did not change at all after silencing of HMGA2 (Supplementary Figure S4A (iii, v)); however, reproducible and significant but weak increases in binding of histone H3 K9ac (and K9me3) were observed in MDA-shHmga2 cells (Supplementary Figure S4A (iv, vi)), which correlate with the observed 8-fold induction of *CDH1* mRNA levels after HMGA2 silencing (Figure 3F). In addition, DNMT3A association to the human *CDH1* promoter remained similar at the promoter region when HMGA2 was knocked down (Supplementary Figure S4A (vii)). This was despite a reproducible and distinct, albeit small, decrease of DNMT3A protein in MDA-shHmga2 cells, compared to MDA-mock cells (Supplementary Figure S4C), indicating that regulation of DNMT3A total levels by HMGA2 was modest in MDA-MB-231 cells. It is therefore possible that the remaining DNMT3A present in MDA-shHmga2 cells could continue to contribute towards epigenetic silencing of the *CDH1* chromatin thus prohibiting very high upregulation of *CDH1* mRNA and protein as observed above (Figure 3F, G). Thus, HMGA2 seems to affect the chromatin landscape on the *CDH1* promoter by perturbing the binding of CTCF and altering the levels of DNMT3A, both events contributing to E-cadherin repression.

## DISCUSSION

EMT is an embryonic program reactivated during carcinogenesis and downregulation of E-cadherin represents a major hallmark of this process (1). In addition to genome-wide expression reprogramming caused by the classical EMT-TFs (4,5), dynamic chromatin modifications also occur during EMT in regulating both epithelial and mesenchymal genes (9,24,37,38). We have previously established a constitutive EMT model by overexpression of HMGA2 in NMuMG cells, which led to the upregulation of Snail and Twist and the downregulation of E-cadherin (12). However, E-cadherin failed to revert when we knocked down Snail and/or Twist from these cells that expressed high HMGA2 levels (17,18). We now demonstrate that this was due to the retainment of repressive epigenetic marks on the *Cdh1* promoter (Figure 1C–E). Here, we investigated an epigenetic and possibly direct role for HMGA2 in the modulation of E-cadherin, a major suppressor of tumour invasiveness (29,30,39). Aberrant HMGA2 levels led to a closed conformation at the *Cdh1* locus as evidenced by DNA methylation and accumulation of H3K9me3 and H3K27me3 repressive histone H3 marks (Figure 1). Furthermore, the modulation of the binding of CTCF (Figure 7), which is a factor that insulates active–inactive chromatin boundaries, enforces the notion that HMGA2 changes the chromatin landscape. We also showed that HMGA2 upregulates DNMT3A expression, and that both HMGA2 and DNMT3A bind to the *Cdh1* promoter (Figure 6). These findings suggest a model whereby HMGA2 and DNMT3A assist the recruitment of other proteins involved in transcriptional repression of *CDH1* (Figure 7H). It will be interesting to analyse possible direct recruitment of co-repressor complex components, such as specific HDACs and proteins that bind to methylated DNA. Finally, treatment with 5-aza-2′-deoxycytidine re-activated the *Cdh1* gene by affecting HMGA2 and DNMT3A associations to its promoter (Figure 6). For clarity reasons the model of Figure 7H does not show the important and established EMT-TFs that directly bind and initiate repression of the *Cdh1* locus, such as Snail or ZEB family members (3,4). The overall evidence that we gather by studying the role of HMGA2 during EMT is that it is a critical chromatin factor that establishes physical contacts and functional cooperation with many key transcriptional regulators of EMT, including Snail and Twist (17,18). Together with these EMT-TFs, HMGA2 seems to be capable to also affect DNA methylation via interaction with DNMT3A, and to affect recruitment of factors like CTCF (Figure 7H). The new results therefore underscore a novel role of HMGA2 on the *Cdh1* gene, since HMGA2 is shown for the first time to be recruited to the *Cdh1* promoter, associate with CTCF and DNMT3A and modulate methylation of CpG sequences on DNA.

TGFβ has previously been shown to contribute to the epigenetic program of EMT (24,37,40). The role of TGFβ in DNMT regulation is context-dependent. It has been demonstrated that TGFβ induces DNA methylation by DNMT1 and imposes an epigenetic memory in immortalized breast epithelial MCF10A cells (40). TGFβ, in cooperation with mitogen activated protein kinases, upregulates DNMTs in prostate cancer (41). The TGFβ effect on *Dnmt3a* (and also *Dnmt3b*) mRNA in NMuMG cells had relatively slow kinetics with a 2-fold induction observed at 24 h post-stimulation (Figure 4E), therefore *Dnmt3a* must be an indirect gene target of the TGFβ signalling pathway. It is plausible that TGFβ would have to first induce HMGA2 or a sequence-specific transcription factor to regulate the DNMT3 family members. The possibility that TGFβ also downregulates DNMT1 at the protein level (Figure 4H) deserves further careful analysis, however, so far our experiments did not favour a role for DNMT1 in the downregulation of E-cadherin during EMT (Figure 4H). Extensive time course experiments of NMuMG cells responding to TGFβ clearly showed that DNMT3A and possibly *Cdh1* promoter methylation may not be required for the early onset of EMT and E-cadherin downregulation (Figure 4G–H), which is in agreement with previously reported bisulphite sequencing analyses of the *Cdh1* promoter in NMuMG cells (37). Sustained TGFβ signaling or even cooperative oncogenic factors may be required for the stable silencing of E-cadherin and the establishment of an irreversible mesenchymal phenotype since withdrawal from long-term TGFβ exposure resulted in complete reversion of the epithelial phenotype and E-cadherin expression (Figure 5A and B). Such oncogenic factors clearly include HMGA2 and Ras as previously established (12,33,42). Interestingly, chromatin-bound factors, such as HMGA2, Snail and DNMT3A, persist over a long period of time, and DNMT3A remained even after withdrawal from TGFβ and complete re-expression of E-cadherin (Figure 5), suggesting that the regulation of DNA methylating enzymes is a slow process during EMT. Alternatively, presence of methylating enzymes, such as DNMT3A, on chromatin, as analyzed by ChIP, is not possible to reveal different mechanisms of regulation of the enzymatic activity of DNMTs during the long period of establishment of EMT or the reversion back to the epithelial state.

Downstream of TGFβ and HMGA2 are Snail and Twist (17, 18), which repress E-cadherin by involving many binding partners that modulate chromatin function (24,37,44,45). The continuous loss of E-cadherin observed in NM-Hmga2-shSnail and NM-Hmga2–shSnail-shTwist stable clones (Supplementary Figure S1A) (18), suggests another layer of regulation by HMGA2 probably acting co-dependently to Snail and Twist. HMGA2 clearly established methylation of the *Cdh1* proximal promoter DNA (Figure 1E) and silencing of Snail and/or Twist was not sufficient to erase this methylation pattern from the promoter (Supplementary Figure S1C and D). The reversion of E-cadherin loss imposed by HMGA2 after treatment with 5-aza, but not after treatment with TSA (Figure 2A), suggests that DNMT activity is required for the methylation of the promoter region, and upon establishment of DNA methylation, blocking the histone deacetylases cannot be as effective due to the terminal state of epigenetic modification achieved. Depending on the cell type or individual tumour type, the specific DNMT member implicated in this mechanism may be different; however, in the NMuMG model where EMT is potently elicited, DNMT3A is the most likely candidate. Moreover, 5-aza treatment of NM-Hmga2 did not significantly affect the histone modifications but instead prevented HMGA2 and DNMT3A from binding to the promoter. The ability of 5-aza to sequester and inhibit DNMTs is known (28), but the mode of action 5-aza exhibits on HMGA2, whether the drug alters the DNA structure to an extent that prohibits HMGA2 binding or directly binds to HMGA2, remains to be elucidated.

HMGA2 lacks transcriptional activity per se, but it exerts its effects through DNA/protein–protein interactions or remodels the chromatin into an open conformation and thus positively or negatively influences gene regulation (43,44). In this study, we show that HMGA2 can silence genes through DNA methylation. The observed interactions between HMGA2 and DNMT3A on chromatin (Figure 6D, E) may explain how DNMT3A is recruited to the *Cdh1* locus as DNMT3A does not exhibit sequence specificity for DNA-binding. It has been reported that Snail recruits DNMTs to the *Cdh1* promoter (37) but DNMT3A was still bound to the *Cdh1* promoter in NM-Hmga2-shSnail and -shSnail-shTwist clones, suggesting the possibility for a Snail-independent mechanism (Supplementary Figure S1F). In addition, DNMT3A is unable to bind to TSS regions enriched in H3K4me3 marks (45) or CTCF-insulated regions (36). This pattern of DNMT3A binding to the *Cdh1* promoter is seen in our analyses since high HMGA2 binding correlates with high DNMT3A binding (Figure 6C) and with low CTCF binding (Figure 7A) and correlates with low content of H3K4me3 (Figure 1C) in the proximal *Cdh1* promoter. HMGA2 disrupts binding of CTCF (Figure 7), a protein involved in maintenance of chromatin topology which insulates tumour suppressor genes (35,46) and which has an inverse occupancy pattern with DNA methylated regions (36). HMGA2, probably via one of its AT-hooks, forms complexes with CTCF and thus prohibits CTCF binding to the *Cdh1* chromatin (Figure 7C and D). We therefore suggest that, with the loss of an active boundary, HMGA2 induces a permissive chromatin structure for optimal DNMT3A binding and subsequent methylating action, and the consequential recruitment of other co-repressor complexes or epigenetic factors (Figure 7H).

Interestingly, despite the knockdown of HMGA2 in MDA-MB-231 breast cancer cells, E-cadherin protein was not re-expressed (Figure 3G). However, *E-cadherin* mRNA was upregulated upon HMGA2 knockdown (Figure 3F) and this coincided with the higher binding of CTCF and Pol II to the *CDH1* promoter (Figure 7A and B). Treatment with 5-aza was sufficient to demonstrate significant E-cadherin protein re-expression in MDA-MB-231 cells (Figure 3G), which implies that genetic manipulation of HMGA2 alone in breast cancer cells is not sufficient to re-establish a demethylated state on chromatin. Moreover, these results reflect the persistency of cancer cells in keeping their invasive traits by exploiting many possible regulatory avenues to silence tumour suppressor genes, such as E-cadherin. Moreover, most cancer cell lines of the basal B subtype expressed high levels of HMGA2 and suffered a loss of E-cadherin (Figure 1F), suggesting that in such tumours, invasiveness promoted by the loss of E-cadherin is a likely event. However, it should be noted that complete suppression of E-cadherin is not an absolute requirement for the invasion of many cancer cells (47).

In agreement with the EMT procurement of cell motility and invasion abilities, HMGA2 rendered cells more invasive and motile (Figure 2). This can be explained only partly by the impact HMGA2 has on E-cadherin expression (Supplementary Figures S2 and S3 and Figure 3). Silencing of HMGA2 in the invasive breast cancer cell model MDA-MB-231 affected, in addition to E-cadherin, the expression of invasive genes, such as *MMP2* and *TNC*, and reduced the rate of motility and infiltration of cells through matrigel (Supplementary Figure S3). However, some mesenchymal genes were unexpectedly upregulated (e.g. *MMP1*) or unchanged (e.g. *fibronectin*, *N-cadherin*, etc.), which suggests that HMGA2 has differential gene targets in different cell models and this must be dependent on the cell-specific chromatin milieu. Despite the evidence provided from genome-wide screens for HMGA2-target genes using transcriptomic microarray platforms (48,49), these studies had focused on different cancer cell models. We currently conduct a ChIP-seq analysis of HMGA2 in breast cancer cells to help identify and better understand the regulation of alternative gene targets by HMGA2 during breast cancer cell invasion. In summary, we have described an epigenetic role for HMGA2 and have begun unravelling how this embryonic chromatin factor could influence the chromatin landscape resulting in multiple outputs by up- and down-regulating genes during EMT and invasion.

## SUPPLEMENTARY DATA

Supplementary Data are available at NAR Online.

SUPPLEMENTARY DATA
