# Supplementary material for: The high mobility group A2 protein epigenetically silences the Cdh1 gene during epithelial-to-mesenchymal transition
Source: Nucleic Acids Res. 2014 Dec 9;43(1):162–78. doi: 10.1093/nar/gku1293 (PMC4288184; doi:10.1093/nar/gku1293)
Supplement: SUPPLEMENTARY DATA [file supp_43_1_162__index.html]

The high mobility group A2 protein epigenetically silences the Cdh1 gene during epithelial-to-mesenchymal transition — SUPPLEMENTARY DATA 

# The high mobility group A2 protein epigenetically silences the *Cdh1* gene during epithelial-to-mesenchymal transition

## SUPPLEMENTARY DATA

**Files in this Data Supplement:**

- SUPPLEMENTARY DATA
