# Supplementary material for: The high mobility group A2 protein epigenetically silences the Cdh1 gene during epithelial-to-mesenchymal transition
Source: Nucleic Acids Res. 2014 Dec 9;43(1):162–78. doi: 10.1093/nar/gku1293 (PMC4288184; doi:10.1093/nar/gku1293)
Supplement: SUPPLEMENTARY DATA [file supp_gku1293_nar-01612-v-2014-File009.pdf]

SUPPLEMENTARY DATA

The High Mobility Group A2 protein epigenetically silences the *Cdh1* gene during epithelial-to-mesenchymal transition

E-Jean Tan, Kaoru Kahata, Oskar Idås, Sylvie Thuault, Carl-Henrik Heldin, and Aristidis Moustakas

SUPPLEMENTARY TABLES

Supplementary Table S1. Oligonucleotides used in quantitative real-time PCR reactions for mRNA analysis.

Gene	Species		Primer Sequence	Product size (bp)	Accession number
Gapdh	mouse	fwd	5'- TGTGTCCGTCGTGGATCTGA -3'	77	NM_008084
		rev	5'- CCTGCTTCACCACCTTCTTGA -3'		
Dnmt1	mouse	fwd	5'- CCAAGCTCCGGACCCTGGATGTGT -3'	373	NM_010066
		rev	5'- CGAGGCCGGTAGTAGTCACAGTAG -3'		
Dnmt3a	mouse	fwd	5'- ATGTGGTTCGGAGATGGCAAG -3'	124	NM_007872
		rev	5'- AGATGGCTTTGCGGTACATGG -3'		
Dnmt3b	mouse	fwd	5'- CAAGGAGGGCGACAACCGTCCATT -3'	221	NM_010068
		rev	5'- TGTTGGACACGTCCGTGTAGTGAG -3'		
Cdh1	mouse	fwd	5'- GACTGTGAAGGGACGGTCAAC -3'	151	NM_009864
		rev	5'- CCACCGTTCTCCTCCGTAGA -3'		
MMP1	human	fwd	5'- CCAAATGGGCTTGAAGCT -3'	100	NM_002421
		rev	5'- gtagcacattctgtccctaa -3'		
MMP2	human	fwd	5'- AGATGCCTGGAATGCCAT -3'	107	NM_004530
		rev	5'- GGTTCTCCAGCTTCAGGTAAT -3'		
TIMP1	human	fwd	5'- GGGGACACCAGAAGTCAACCAGA -3'	400	NM_003254
		rev	5'- CTTTTCAGAGCCTTGGAGGAGCT -3'		
TIMP3	human	fwd	5'- CTACACCATCAAGCAGATGAAGAT -3'	455	NM_000362

		rev	5’- TCCAGGGGTCTGTGGCATTGAT -3’		
TNC	human	fwd	5’- GCTCAACCATCACTGCCAAGT -3’	81	NM_002160
		rev	5’- CAGTTTCCGACTGAACCTCAGTAG -3’		
KLF4	human	fwd	5’- CGACGCGCTGCTCCCATCTT -3’	97	NM_004235
		rev	5’- CCGCCAGCGGTTATTCGGGG -3’		
CDH1	human	fwd	5’- TACGCCTGGGACTCCACCTA -3’	101	NM_004360
		rev	5’- CCAGAAACGGAGGCCTGAT -3’		
HMGA2	human	fwd	5’- GACGTCGGGCATTCATATAGG -3’	105	NM_003483
		rev	5’- TTGGTGTTCTAAACAGAGGATTCACT -3’		
GAPDH	human	fwd	5’- GGAGTCAACGGATTTGGTCGTA -3’	78	NM_002046
		rev	5’- GGCAACAATATCCACTTTACCA -3’		

Supplementary Table S2. Oligonucleotides used in quantitative real-time PCR reactions for chromatin immunoprecipitation assays or in restriction enzyme methylation assays.

Position relative to TSS*		Primer Sequence	Product size (bp)
mouse *Cdh1* -108/+3 promoter	fwd	5’- GGCCCTGCAGTTCCTTGGCT -3’	111
	rev	5’- AGTGAGCAGCGCAGAGGCTG -3’	
human *CDH1* -78/+26 promoter	fwd	5’- AGGTGAACCCTCAGCCAATCAGCG -3’	105
	rev	5’- AGGTGCTTTGCAGTTCCGACGC -3’	

*TSS = transcription start site

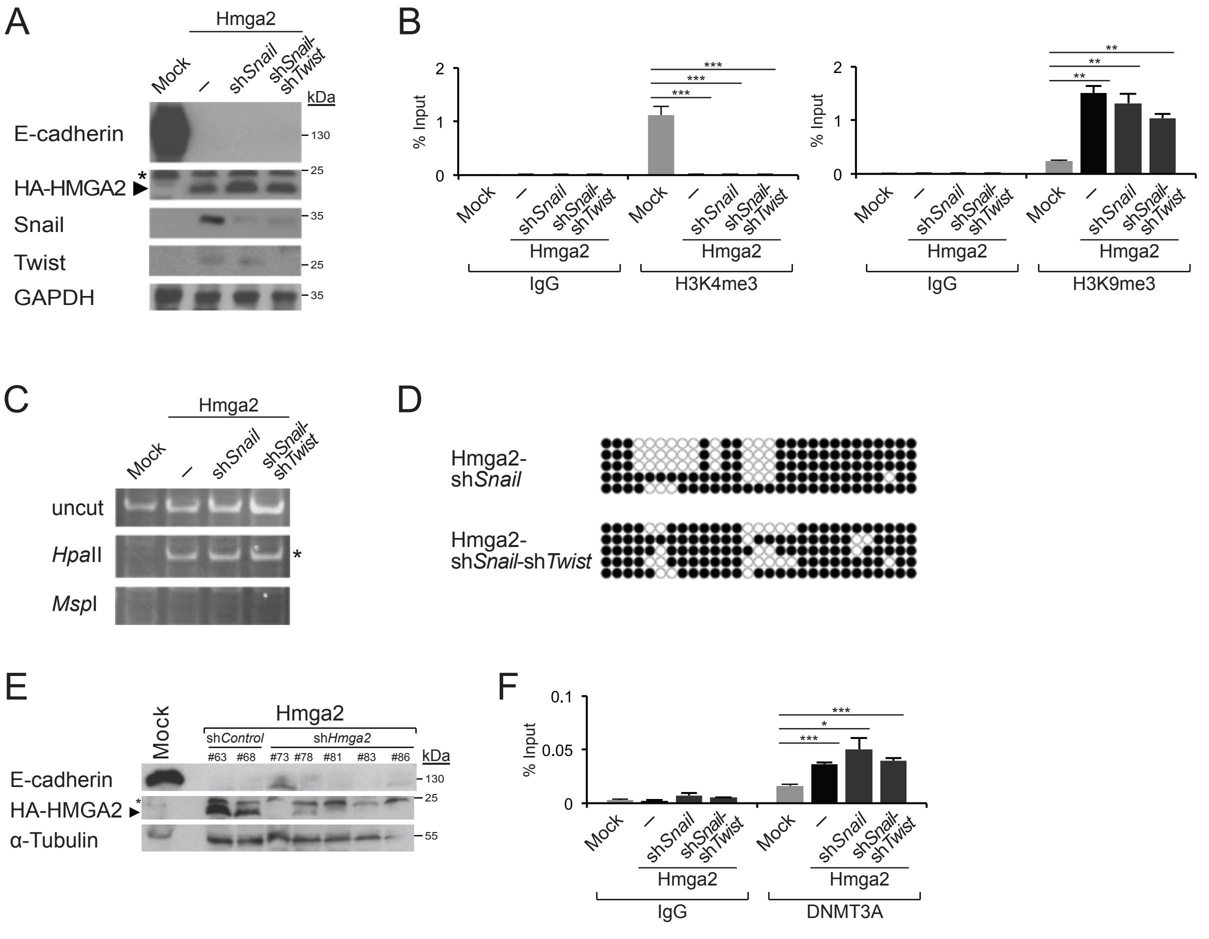
A
Hmga2
Mock
—
shSnail
shSnail-shTwist
kDa
E-cadherin
130
HA-HMGA2
*
25
Snail
35
Twist
25
GAPDH
35
B
% Input
IgG
H3K4me3
H3K9me3
C
uncut
HpaII
MspI
D
Hmga2-shSnail
Hmga2-shSnail-shTwist
E
shControl
shHmga2
#63 #68 #73 #78 #81 #83 #86
α-Tubulin
55
F
DNMT3A

Figure S1: Epigenetic repression of the *Cdh1* promoter by HMGA2 is independent of Snail and Twist. (A) Immunoblots for E-cadherin, HA-tagged HMGA2, Snail and Twist in NM-Mock and NM-Hmga2 and its derivative stable clones (sh*Snail* and sh*Snail*-sh*Twist*). GAPDH serves as loading control. (B) ChIP-qPCR assays of active H3K4me3 and repressive H3K9me3 histone marks on the *Cdh1* promoter in cells described in panel A. (C) *Hpa*II-*Msp*I digestion-methylation assay of the *Cdh1* promoter in cells described in panel A. The PCR product was subjected to agarose gel electrophoresis and bands observed after *Hpa*II-digestion indicate that the amplified DNA was methylated (asterisk). (D) DNA methylation status of the *Cdh1* promoter in NM-Hmga2 cells depleted of Snail or both Snail and Twist, was analysed by bisulphite sequencing of the *Cdh1* promoter region shown in Figure 1A, and 5 out of 10 clones of each cell line are shown here. White and black circles represent unmethylated and methylated CpG sites respectively. (E) Immunoblots for E-cadherin, HA-HMGA2 (arrowhead) and α-tubulin in NM-Hmga2 cells depleted of HMGA2. Asterisk indicates an unspecific band. (F) ChIP-qPCR analysis with IgG or DNMT3A antibody on the *Cdh1* promoter in cells described in panel A.

Supplementary Figure S2 Tan *et al.*

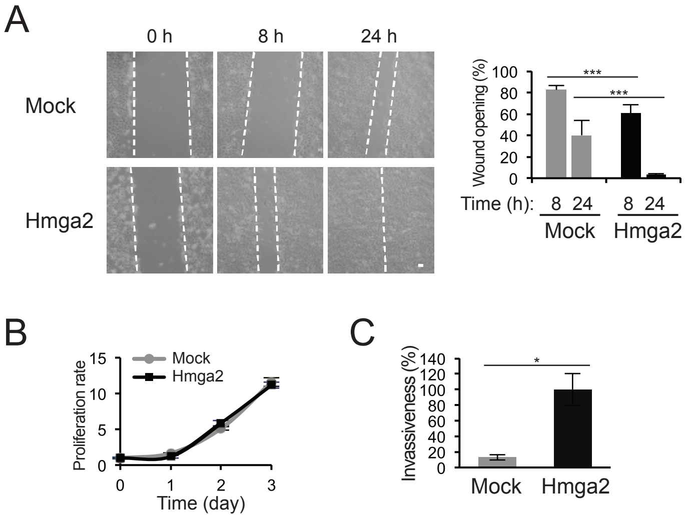

Figure S2: HMGA2 promotes migration and invasion. (A) Light microscopic images of NM-Mock and NM-Hmga2 cells in a wound healing assay at 0, 8 and 24 h after the wound. *Scale bar*, 50 µm. Bar graph shows wound opening at 24 h as a percentage of original wound opening at 0 h (right panel; mean ± SD from 9 fields). (B) MTS assay was performed over 3 days to measure cell proliferation rate in NM-Mock and NM-Hmga2 cells (mean ± SD from 5 replicates). The proliferation rate is measured in arbitrary units (relative, normalised values). (C) Invasion ability of NM-Mock and NM-Hmga2 cells were assayed using a Matrigel transwell assay. The bar graph shows invasion rate relative to NM-Hmga2 cells, which is set to 100% (mean ± SD values from triplicates). The experiments were done at least twice.

Supplementary Figure S3 Tan *et al.*

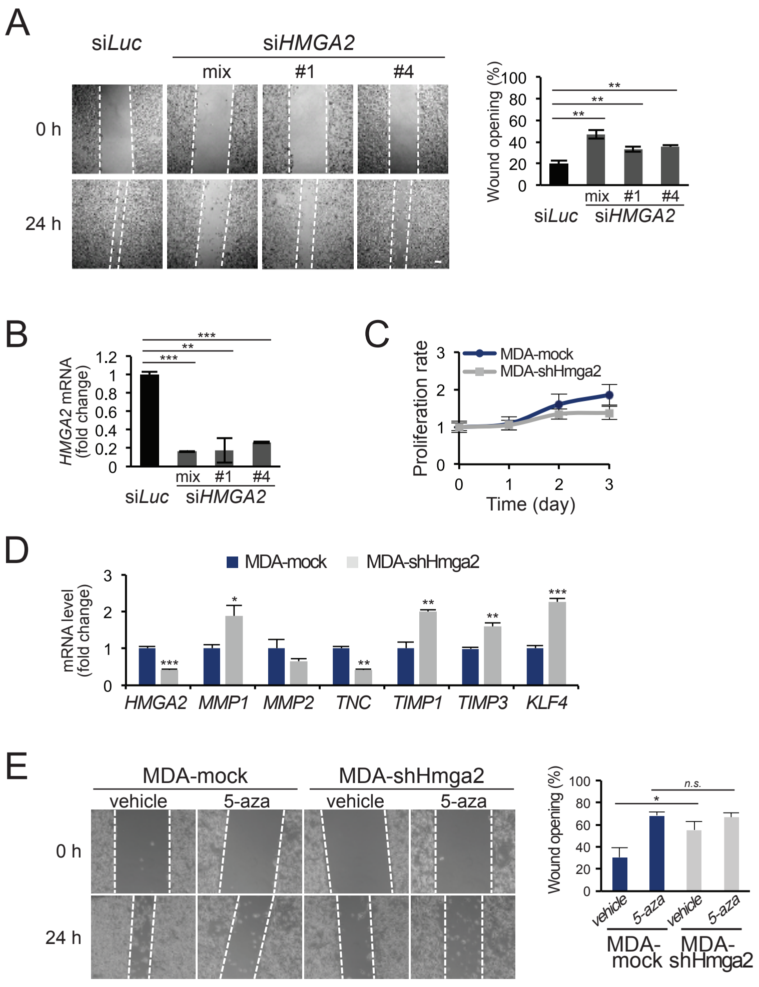

Figure S3. HMGA2 promotes cell migration in breast cancer cells. (A) Wound healing assay in MDA-MB-231 cells transiently transfected with control siRNA (si*Luc*) or two distinct siRNAs against HMGA2 (#1, #4) and their combination (mix). Bar graph represents the percentage of wound opening at 24 h as a percentage of original wound opening at 0 h (right panel; mean ± SD from three independent measurements). (B) Knockdown efficiency of HMGA2 in cells used in the wound healing assay in panel A and analysed by qPCR (mean ± SD from triplicate values). (C) MTS assay was performed over the course of 3 days, as described in Supplementary Figure S2B, to measure cell proliferation rate in MDA-mock and MDA-shHmga2 cells (mean ± SD from 5 replicates). (D) Expression levels of genes, *HMGA2, MMP1, MMP2, TNC, TIMP1, TIMP3 and KLF4*, normalised to *GAPDH* mRNA levels in MDA-mock and MDA-shHmga2 cells. Expression levels in MDA-mock cell are normalised to 1. (E) Wound healing assay of MDA-mock and MDA-shHmga2 cells treated with vehicle or 5-aza, as described in Figure 2C. Bar graph shows wound opening at 24 h as a percentage of original wound opening at 0 h (right panel; mean ± SD from 9 fields).

Supplementary Figure S4 Tan *et al.*

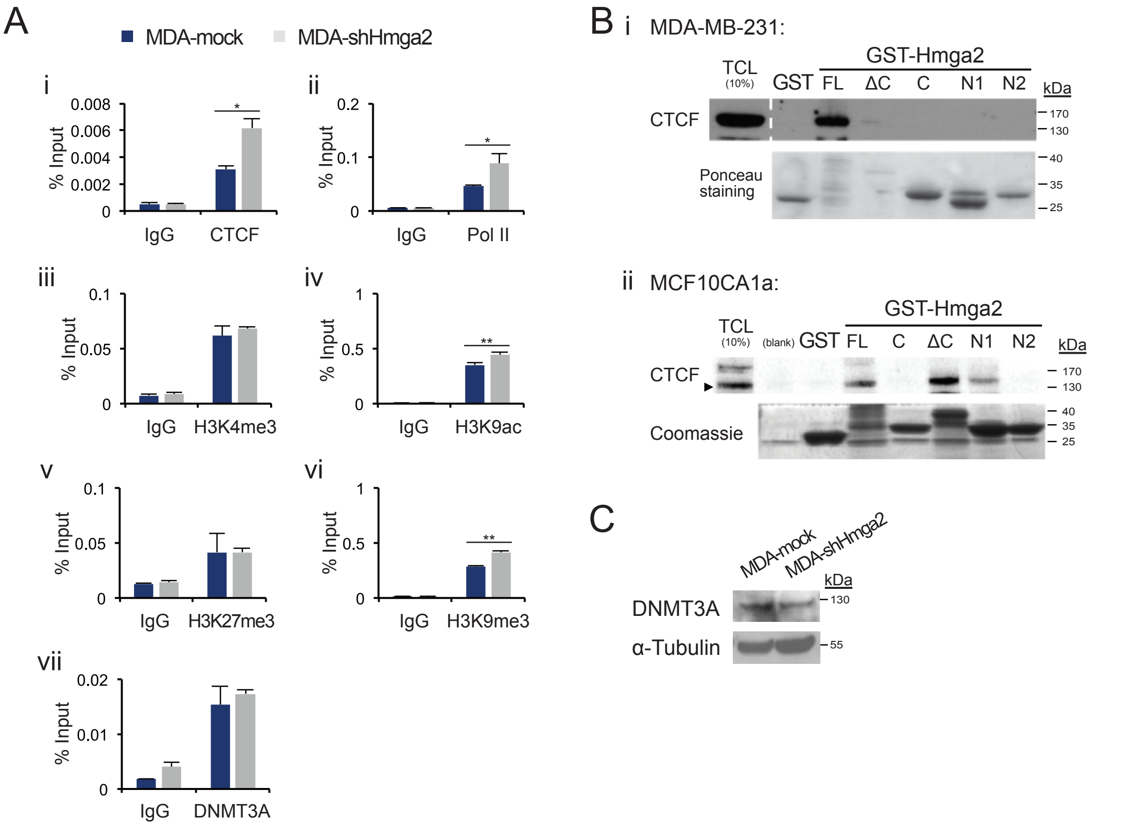

Figure S4. HMGA2 interacts with CTCF in breast cancer cells. (A) ChIP-qPCR analyses of CTCF (*i)*; Pol II (*ii*); H3K4me3 (*iii*); H3K9ac (*iv*); H3K27me3 (*v*); H3K9me3 (*vi*); and DNMT3A (*vii*) binding on the *CDH1* promoter in MDA-mock and MDA-shHmga2 cells. (B) GST-HMGA2 and its deletion mutants were used in a pull-down assay with parental MDA-MB-231 (*i*) or MCF10CA1a (*ii*) cell extracts and immunoblotted for CTCF. Input of GST fusion proteins used in the pull-down were visualised by Ponceau-S or coomassie brilliant blue staining. *FL*, full-length; *ΔC*, deletion of C-terminal; *N1*, N-terminal 1; *N2*, N-terminal 2; *C*, C-terminal; *TCL*, total cell lysate. (C) DNMT3A and GAPDH protein levels in MDA-control and -shHmga2 cells.
